# Four-Year Trends in Sleep Duration and Quality: A Longitudinal Study Using Data from a Commercially Available Sleep Tracker

**DOI:** 10.2196/14735

**Published:** 2020-02-20

**Authors:** Rebecca Robbins, Mahmoud Affouf, Azizi Seixas, Louis Beaugris, George Avirappattu, Girardin Jean-Louis

**Affiliations:** 1 Division of Sleep and Circadian Disorders Harvard Medical School Boston, MA United States; 2 Brigham and Women's Hospital Boston, MA United States; 3 Mathematical Sciences Kean University Union, NJ United States; 4 Center for Healthful Behavior Change Department of Population Health NYU School of Medicine New York, NY United States

**Keywords:** big data, sleep health, fitness tracker, mHealth

## Abstract

**Background:**

Population estimates of sleep duration and quality are inconsistent because they rely primarily on self-reported data. Passive and ubiquitous digital tracking and wearable devices may provide more accurate estimates of sleep duration and quality.

**Objective:**

This study aimed to identify trends in sleep duration and quality in New York City based on 2 million nights of data from users of a popular mobile sleep app.

**Methods:**

We examined sleep duration and quality using 2,161,067 nights of data captured from 2015 to 2018 by Sleep Cycle, a popular sleep-tracking app. In this analysis, we explored differences in sleep parameters based on demographic factors, including age and sex. We used graphical matrix representations of data (heat maps) and geospatial analyses to compare sleep duration (in hours) and sleep quality (based on time in bed, deep sleep time, sleep consistency, and number of times fully awake), considering potential effects of day of the week and seasonality.

**Results:**

Women represented 46.43% (1,003,421/2,161,067) of the sample, and men represented 53.57% (1,157,646/2,161,067) of individuals in the sample. The average age of the sample was 31.0 years (SD 10.6). The mean sleep duration of the total sample was 7.11 hours (SD 1.4). Women slept longer on average (mean 7.27 hours, SD 1.4) than men (mean 7 hours, SD 1.3; *P*<.001). Trend analysis indicated longer sleep duration and higher sleep quality among older individuals than among younger (*P*<.001). On average, sleep duration was longer on the weekend nights (mean 7.19 hours, SD 1.5) than on weeknights (mean 7.09 hours, SD 1.3; *P*<.001).

**Conclusions:**

Our study of data from a commercially available sleep tracker showed that women experienced longer sleep duration and higher sleep quality in nearly every age group than men, and a low proportion of young adults obtained the recommended sleep duration. Future research may compare sleep measures obtained via wearable sleep trackers with validated research-grade measures of sleep.

## Introduction

### Background

Sleep is essential to a variety of domains of health, including weight management, mood regulation, and longevity [1-3]. National survey research purports that 25% of adults in the United States do not meet the recommended duration of sleep, which is 7 to 9 hours; however, these findings rely on self-reported data [4], which has been shown to vary widely from objectively monitored sleep [5]. The increasingly low-cost technologies embedded in mobile phone and wearable devices provide users with the ability to track their behaviors, such as sleep [6-9]. Sleep tracking with commercially available technology has become popular among the general population in recent years, which presents researchers with an opportunity to analyze the big data captured by these trackers and examine trends in population sleep health.

Behavioral monitoring or the ability to document and reflect on one’s own behavior is an essential part of health behavior according to prominent theories. Specifically, cognitive behavioral therapy [10] and self-regulation theory [11] articulate the importance of behavioral modeling for achieving a desired health outcome. Sleep tracking on a mobile device could be viewed as a form of behavioral monitoring. Although sleep-tracking devices were initially very crude approximations of sleep duration, several of the consumer-facing tracking devices now offer robust visualizations, often including sleep staging and overall sleep performance in the form of a score summarizing such components as sleep quality [12,13]. Indeed, an emerging literature shows the results of studies comparing sleep output from consumer-facing sleep trackers with polysomnography, the gold standard for sleep measurement [14,15].

Sleep tracking via mobile technology is increasingly available to our global population. Research has shown that ownership of smartphones, which feature components that afford the ability to sleep track, such as accelerometers, is reported by approximately half of those living in developing areas of the world and by as many as 90% of those living in developed areas [16]. National data in the United States show approximately one-third of the population report regularly using a smartphone or other devices to track their sleep [17], suggesting sleep tracking is a common practice among individuals in the United States.

Another area of research has examined the design of apps for aiding in sleep disorder symptom identification, such as snoring as a risk factor for sleep apnea [18,19]. Moreover, 1 study analyzed a large volume of sleep tracker data and compared sleep before and after major political events [20]; yet otherwise, no research has explored trends over time in sleep data captured by a sleep tracker app or device.

### Objectives

We undertook an analysis of 4 years of data from a popular wearable sleep tracker to examine patterns of use among users in New York City.

We explored potential seasonal differences in sleep as captured by the sleep tracker. We also examined differences in sleep during the weeknight and weekend nights and contrasted the potential effects of age, sex, and life stage.

## Methods

### Study Overview

We performed historical research using data obtained from Sleep Cycle app. This app is a sleep tracker that uses accelerometer and auditory input from a smartphone device to detect sleep duration and stages. Sleep Cycle is a low-cost app (US $1) available for iPhone and Android operating platforms. This analysis was conducted at the New York University School of Medicine and Kean University. The data used in this analysis were obtained directly from the Sleep Cycle app.

### Participants

Eligibility criteria for this study included living in a major urban center (New York City) and age 13 years or older. Location was detected using built-in GPS, which allows the phone to detect the users and their location in the form of latitude and longitude coordinates. We requested data for users living within the latitude and longitude that signify New York City and its 5 boroughs for this analysis.

We chose New York City as a location for several reasons. First, we chose 1 geographic location, as this would avoid having to control for varying weather, light, noise, or other environmental factors if we were to compare multiple urban centers. Second, inhabitants of the central region of New York City (Manhattan) include those with a higher average income, whereas the outer regions have inhabitants with a different socioeconomic profile. Therefore, New York City provides a backdrop for examining sleep across different socioeconomic regions in a major urban center.

Data were obtained in an anonymous and aggregated dataset from the developers of the tracker without personal identifiers. Users provided their email address and name to create an account. These sensitive data were removed from the dataset we received and replaced with a random number. According to the Sleep Cycle app’s privacy policy, users consented to provide access to their location while using the tracker. We requested all data on users based in New York City over the past 4 years for this analysis. We analyzed 2,161,067 nights of data, which were provided by 160,963 participants during a 4-year period. The app developers cautioned that a drop in users in 2016 is observed because of a change in the company’s privacy policy.

### Measures

Users of the sleep tracker place their smartphone device either at their bedside or on their mattress during their sleep, where the app is able to detect motion and sound as inputs. These inputs were scored using proprietary algorithms. Users received detailed statistics and visuals on their sleep from the night before. The app developers maintain that the algorithm to score sleep duration and quality is identical when users track their sleep from either their mattress or bedside.

### Sleep Duration

This analysis was based on sleep duration and sleep quality data recorded by the Sleep Cycle app. Sleep duration was captured objectively and displayed in hours and minutes. Sleep duration was collected for weeknight and weekend night. We scored the sleep duration data using the National Sleep Foundation recommendations, which include *recommended*, *may be appropriate*, and *not appropriate* ranges for different age groups. For instance, *recommended* duration for teenagers (aged 13-17 years) was 8 to 10 hours, *maybe appropriate* duration for teenagers was either 7 to 8 hours or 10 to 11 hours, and *not appropriate* duration for teenagers was either less than 7 hours or more than 11 hours.

### Sleep Quality

This analysis used sleep quality as derived by the Sleep Cycle app. The app reports sleep quality as a score of the overall efficiency of the sleep using an algorithm based on time in bed, deep sleep time, sleep consistency, and amount of times fully awake. Sleep quality scores range from low (0) to high (100). Drawing on the sleep efficiency index, we conceptualized sleep quality scores of 85 and above as good quality and those below 85 as poor sleep quality [21].

Demographic factors are obtained for each user and self-reported in their profile. These data were obtained along with sleep duration and quality for each user. Demographic variables included age and sex. We eliminated individuals aged younger than 13 years and older than 85 years. In addition, each night of sleep included a date and time stamp. Consequently, we created variables to distinguish between sleep during summer, winter, spring, and autumn.

### Analysis

We computed descriptive statistics for sleep duration by independent variables (weeknight vs weekend night, age, sex, and seasonality). Similarly, we computed descriptive statistics for sleep quality by weeknight versus weekend night, age, sex, and seasonality. We used logistic regression to examine differences in sleep duration and quality by independent variables. We created graphical matrix representations of data to graphically display the differences in sleep duration and quality based on weeknight versus weekend night, age, sex, and seasonality. We compared sleep duration and quality by weeknight (vs weekend night), age, sex, and seasonality with analysis of variance or Chi-square and reported the *P* value to indicate significant differences in sleep parameters.

Finally, we created a heat map, consistent with previous research [22], whereby red indicated greater concentration of users who demonstrated *not recommended* sleep duration on a day chosen at random from the 4 years of data. Per the National Sleep Foundation recommendations, this cutoff varied by age group. Specifically, users were marked in red if their sleep for the night chosen at random did not meet the criteria for *recommended* in their respective age group. We matched these individuals, using the latitude and longitude data obtained from the app, to a geographical map of New York City to visually examine the concentration of *not recommended* sleep duration in New York City. All analyses were performed in R software.

## Results

Of the overall nights studied, females contributed 46.43% (1,003,421/2,161,067) of nights in this analysis, and males contributed 53.57% (1,157,646/2,161,067) of nights. These proportions were similar to the sex profile of the sample (n=160,963). Females represented 72,862 out of 160,963 (45.27%), and males represented 88,122 out of 160,963 (54.75%). There was a relatively even breakdown between nights in this analysis across the 4 years, although the highest proportion of nights (652,391/2,161,067; 30.19%) came from 2017. Overall, weeknights (1,299,037/2,161,067; 60.07%) outnumbered weekend nights tracked (862,030/2,161,067; 39.89%). Individuals aged 26 to 64 years provided the most nights of any age group (1,298,200/2,161,067; 60.07%). All comparisons between demographic factors were significant at the *P*<.05 level (see Table 1).

**Table 1:** General characteristics, including demographic factors (age and sex), and weeknight versus weekend night sleep recording (n=2,161,067 nights of sleep tracking).

Variable		Total, n (%)	Female, n (%)	Male, n (%)		*P* value
Total		2,161,067 (100.00)	1,003,421 (46.43)	1,157,646 (53.57)		<.001
Unique users		160,963 (100.00)	72,862 (45.27)	88,122 (54.75)		<.001
**Yearly records**						
	2015	459,639 (21.27)	209,828 (45.65)	249,811 (54.35)		—^a^
	2016	581,709 (26.92)	267,202 (45.93)	314,507 (54.07)		—
	2017	652,391 (30.19)	305,734 (46.86)	346,657 (53.14)		—
	2018	467,328 (21.62)	220,657 (47.22)	246,671 (52.78)		—
**Weeknight versus weekend night**					<.001	
	Weekday	1,299,037 (60.11)	596,447 (45.91)	702,590 (54.09)		
	Weekend	862,030 (39.89)	406,974 (47.21)	455,056 (52.79)		
**Age group (years)**					<.001	
	Teens (13-17)	97,156 (4.50)	55,389 (57.01)	41,767 (42.99)		
	Young adults (18-25)	739,423 (39.89)	358,584 (48.50)	380,839 (51.50)		
	Adults (26-64)	1,298,200 (60.07)	578,676 (44.58)	719,524 (55.42)		
	Older adults (65-84)	26,288 (1.22)	10,772 (40.98)	15,516 (59.02)		

^a^Not applicable.

The average age of the sample was 31.0 years (SD 10.62). Overall, the average sleep duration was 7.1 hours (SD 1.4), and the average sleep quality was 72.3 (SD 14.2). Women demonstrated overall slightly longer sleep duration (mean 7.3, SD 1.4) than men (mean 7.0, SD 1.3). Women also demonstrated higher sleep quality (mean 73.4, SD 14.1) than men (mean 71.3, SD 14.2).

Teenagers met sleep recommendations on 26.23% of nights (25,491/97,156). Young adults met sleep recommendations on 24.61% of nights (364,065/1,478,846), and adults met sleep recommendations on 51.91% of adults (673,947/1,298,200). Older adults met sleep recommendations on 33.91% (8914/26,288 nights). Good quality sleep was observed on 21.56% (20,951/97,156 nights) among teenagers, 19.00% (140,428/739,423 nights) among young adults, 19.26% (249,970/1,298,200 nights) among adults, and 23.81% (6260/26,288 nights) among older adults. Supplemental tables provide full descriptive statistics (Multimedia Appendices 1 and 2).

**Figure 1:**
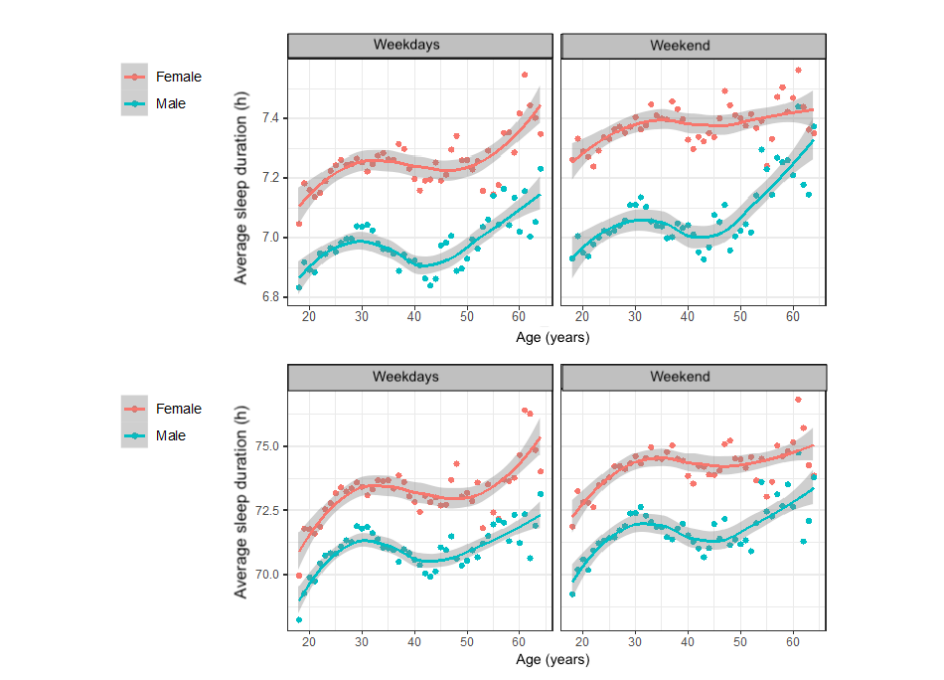
Sleep duration and sleep quality by sex on weekdays and weekends (n=2,161,067 nights of sleep tracking).

Figure 1 shows sleep duration on weekend night and weeknight by age and sex. The gray shading represents the 95% CIs. The graph displays higher sleep duration and higher sleep quality among women than among men at each age group. These graphs display waxing and waning sleep duration and quality over age group.

**Figure 2:**
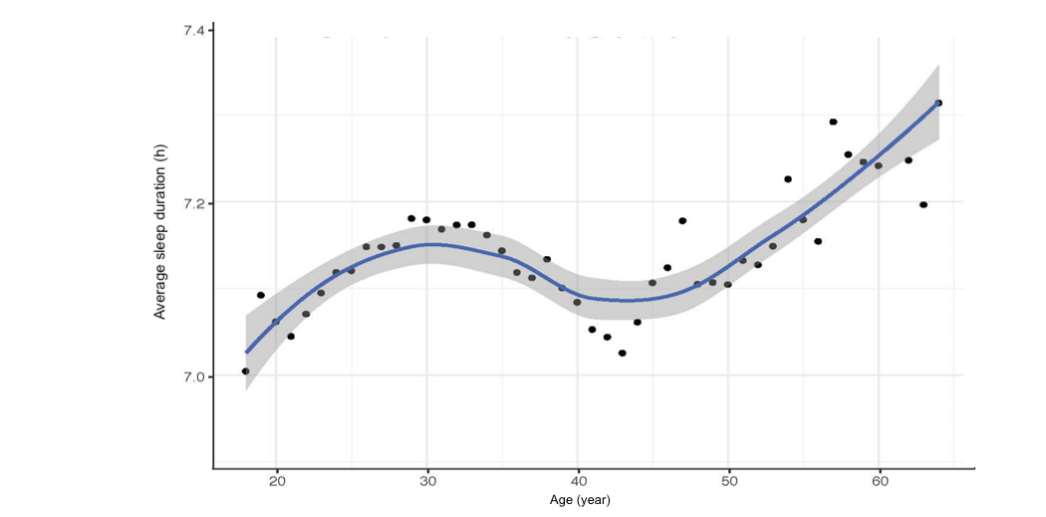
Average sleep duration from age 18 to 64 (n=2,161,067 nights of sleep tracking).

Figure 2 shows sleep duration across the lifespan, from age 18 to 64 years. We did not include the lower age limits (individuals aged <18 or >64 years), as these age groups were unbalanced and led to skewness. As shown in this graph, sleep duration declined sharply among teenagers until approximately age 20 years. Sleep duration hovers around 7.2 hours between age 20 and 35 years, declined slightly around age 40 years, and then increased at age 60 years until age 84 years.

**Figure 3:**
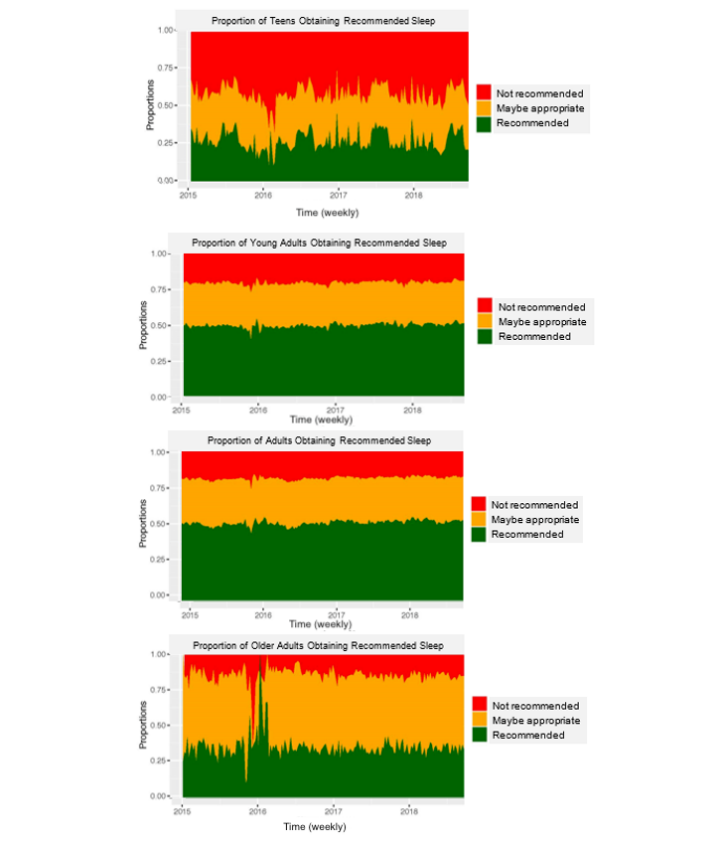
Proportion adhering to sleep recommendations by age group over the four years of the study (n=2,161,067 nights of sleep tracking).

Figure 3 shows sleep recommendations by age group over time. Among teens, there was a large proportion of individuals who obtained recommended sleep duration (<7 hours). Among older adults, the largest proportion of individuals was in the *may be appropriate* category of 5 to 7 hours. Among young adults, the majority appeared to obtain the recommended 7 to 9 hours, as are adults. The largest proportion of adults demonstrated recommended sleep duration across the 4 years. Importantly, these images display the inconsistency in sleep schedules kept by teens and older adults. Although sleep is relatively unchanged over the year, teens and older adults vary widely from week to week in their sleep timing. 

**Figure 4:**
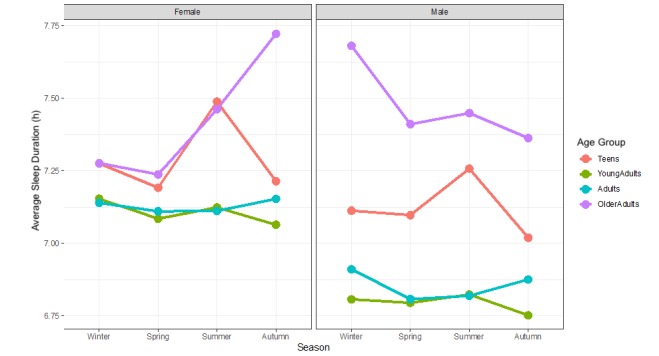
Sleep duration shown by age group and sex across the seasons (n=2,161,067 nights of sleep tracking). AgeGR: Age group.

Regarding seasonality, Figure 4 shows all teenagers (male and female) obtained the longest sleep duration during the summer months. Young adult and adult males and females had somewhat similar patterns, with more sleep during winter and less sleep during spring and summer. Older adult males and females had nuanced patterns, whereby older males slept longest in winter and shortest in the autumn, and older females slept longest in autumn and shortest in the spring.

**Figure 5:**
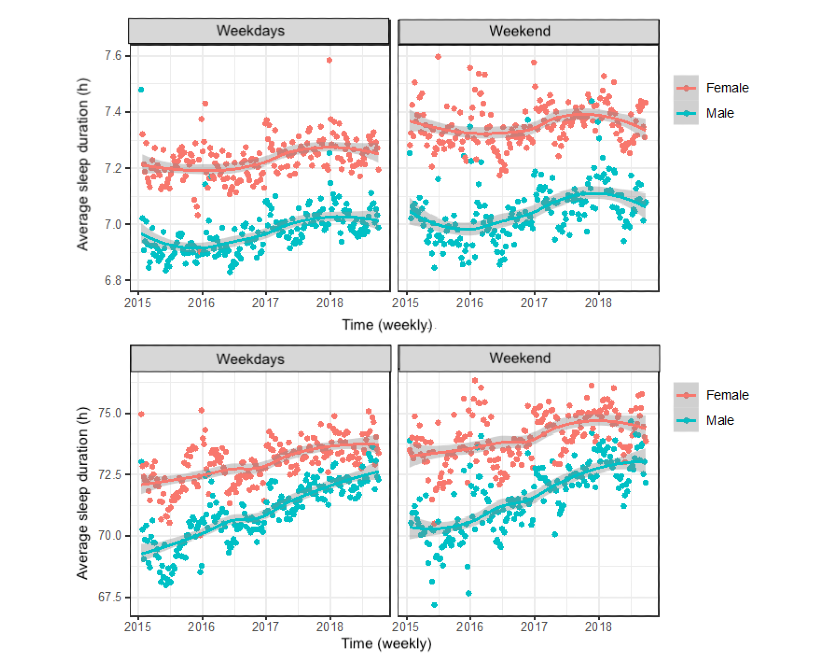
Sleep duration and quality by sex and between weekend and weeknight over the four years of the study (n=2,161,067 nights of sleep tracking).

On the one hand, regarding change in overall sleep duration or quality across the 4 years, sleep duration was lowest for both men and women in 2016, highest in late 2018 and early 2018, and then declined again after the year 2018 as shown in Figure 5. On the other hand, sleep quality trend analysis showed an overall steady increase since 2015, which was the lowest level from the standpoint of sleep quality for men and women.

**Figure 6:**
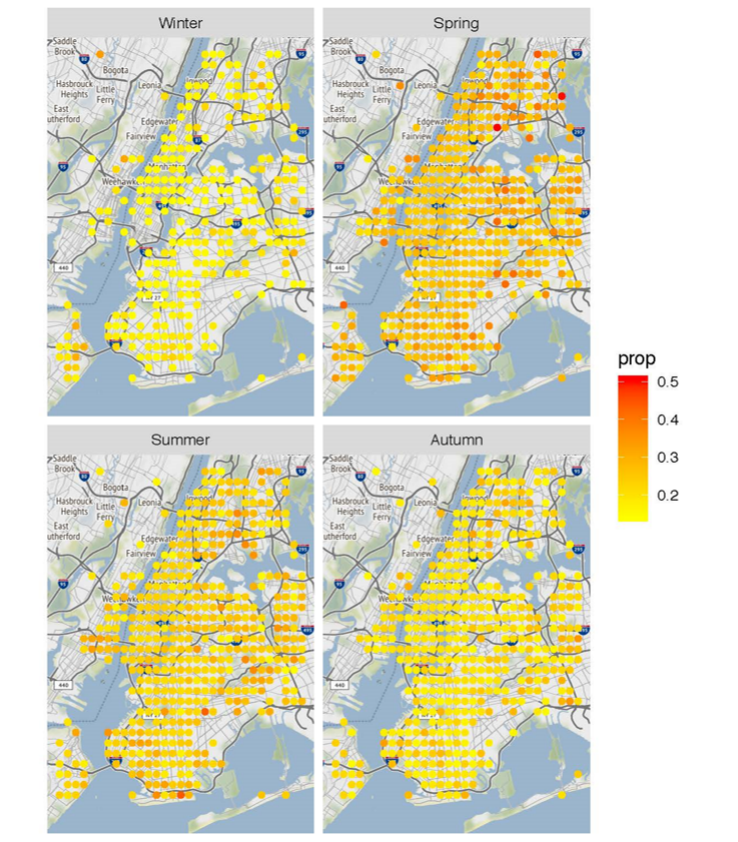
Heat map depicting concentration of 100 users or more in a specific geographical area who demonstrate ‘not recommended’ sleep duration.

The heat map in Figure 6 prevalence of *not recommended* sleep duration graphically across the map of New York City. Sleep data are displayed from 1 night chosen at random from each of the 4 seasons. The map demonstrated a concentration of *not recommended* sleep during the spring season. There is also preliminary support shown in this graph for better sleep outcomes in central New York City (Manhattan) and greater prevalence of *not recommended* sleep duration in outer regions where there is more crime and poverty.

## Discussion

### Principal Findings

Sleep tracking may confer benefits such as increasing motivation for healthy changes to sleep routines among users of sleep trackers [6-9]. Furthermore, sleep tracking is reported using a smartphone or other devices by approximately one-third of the population in the United States [17]. Although researchers articulate the need for validation of commercially available apps and sleep trackers [15,23], we emphasize the need to understand the nature of the data that users of popular sleep trackers receive but also the opportunity to identify a signal in population-level sleep health vis-à-vis the data provided from commercially available sleep trackers. In this study, we analyze big data from users of a popular sleep tracker to illuminate trends in sleep on this platform over 4 years and over 2 million nights.

First, our results showed that women, according to the sleep-tracking app, experience longer sleep duration and higher sleep quality in nearly every age group than men. This finding is somewhat perplexing, as previous research has shown women are sleepier than men (which might indicate poorer sleep quality) and take longer to fall asleep, and older women sleep less (approximately 20 min less) than men [24]. Some previous studies point to the increased household responsibilities many women shoulder disproportionately compared with men, which would systematically detract from sleep time, as a reason for shorter and lower quality sleep among women compared with men [25]. However, our results showed the contrary that women experienced longer sleep duration and higher quality sleep as collected by the app than their male counterparts. We postulate the perplexing finding that women obtain longer sleep and higher sleep quality because of several factors, but notably, there is a chance that the sleep tracker may be poorly suited for detecting sleep among women. For instance, women may have a lighter frame, and therefore, the tracker is less able to detect their movements. It is also possible that cosleeping couples may confound the tracker and its ability to efficiently capture sleep among both individuals.

Second, our study showed a low proportion of teenagers (25,491/97,156; 26.23% of individuals aged 13-17 years) obtained recommended sleep duration. In addition to documenting insufficient sleep among teenagers, our research also shows the prevalent inconsistent sleep schedules maintained by many teenagers. These findings are consistent with previous research that has demonstrated clear reductions in sleep time during the teenage years [26-28]. Research shows the significant barriers teenagers face to sleep health, including social pressures and academic responsibilities, which place teenagers at a significant disadvantage when it comes to their ability to obtain sufficient sleep. Furthermore, this research also highlights structural barriers, such as early school start times, that compete with teenager physiological preference for later bedtimes and result in teenagers extending sleep on the weekend nights, which can introduce social jetlag and circadian desynchrony hindering sleep and overall health [29]. Our research further emboldens the need for public health and policy efforts to address to the issue of poor sleep health among teenagers.

Third, our research examined seasonal patterns in sleep duration between summer, winter, spring, and autumn. Interestingly, there were unique patterns of sleep by season for each age group and sex, with the exception. On the one hand, among young adults and adults, both sexes slept longest during winter. Teenagers, on the other hand, perhaps because of summer vacation and being free of academic and school responsibilities, slept most during the summer months. Interestingly, older adult males had almost entirely different patterns of sleep duration compared with female older adults.

Regarding sleep duration and quality over the 4 years of analysis in New York City, we found sleep duration and quality were lowest around 2015 and early 2016, rising steadily to 2018, at which time there appears to be a plateau. One could likely look to national and international events at these times to explain the slight decline in 2016. For instance, the advent of the gig economy and trend toward project-based work may have led some individuals to shorten sleep or sleep less because of increased occupational burden and less stability that is common among these occupational categories [30]. Overall, sleep duration and quality are higher across the age groups on weekend nights than on weeknights. This is also consistent with previous literature, showing that individuals tend to sleep longer on the weekend nights [31]. Our research similarly found overall longer sleep on weekend nights (compared with weeknights).

Our results provide a comprehensive assessment of 4 years of data and over 2 million nights of sleep. We identified trends in seasonality, age, and sex. In so doing, we provide a compelling case for the issues regarding insufficient sleep among teenage populations. Our geospatial analyses revealed a higher concentration of users receiving *not recommended* sleep duration in outer boroughs of New York City, suggesting sleep that is *recommended duration* may be more common among higher income inhabitants of central New York City.

### Strengths and Limitations

Although the strengths of our analysis included big data used to perform the analyses of sleep data from a large population of users over several years, limitations must be noted. First, we analyzed 1 major metropolitan area. Results will differ in different geographical regions. It must also be noted that although ownership of smartphones that allow this type of tracking is high among developed countries, fewer than half of certain populations report access to smartphones. Consequently, our results regarding sleep duration and quality may be disproportionately represented by high-income populations with access to these technologies. We note that the dataset provided included limited details on the study sample. We received only age, sex, sleep duration, and quality. Future research may aim to include additional variables, such as race and ethnicity or health conditions, for a greater understanding of sleep between a richer array of demographic and health variables.

Our results were performed on the summary scores for sleep duration and quality as reported by the developers of the Sleep Cycle app. The authors of this study did not have access to the algorithm used by the developers to detect, measure, or analyze sleep. Furthermore, there have not been any published studies validating the methodology used by the developers of the Sleep Cycle app with established measures of sleep assessment, such as either wrist-based actigraphy or polysomnography. Previous researchers have emphasized the need for validation of sleep tracking devices such as Sleep Cycle [14,15]. Therefore, it is possible that sleep duration is scored using similar metrics to those used for measuring sleep quality. Therefore, there is a possibility that sleep duration and quality data are correlated with one another. Finally, there is a possibility that different types of cell phone hardware and accelerometer technology may produce differences in sleep as detected by the app.

### Future Research

Our study identified several compelling avenues for future research. First, interventions could be designed to target the specific barriers (eg, insufficient sleep or poor-quality sleep) as reported by users of apps such as that studied in this paper. In addition, researchers and developers of sleep-tracking technology could collaborate on the sleep duration and quality algorithms to ensure concordance with the gold standard in sleep research (ie, sleep diary, actigraphy, or polysomnography). Finally, future research may also examine the effect of exposure to sleep-tracking apps. For instance, although we know sleep tracking is increasingly common, we know less about the effects of its exposure, the duration of adherence to the devices, and how helpful (or perhaps harmful) output regarding sleep may be for individuals, particularly those suffering from disorders, such as insomnia or sleep apnea. Among these patient populations, sleep tracking may be ill advised as a source of worry about sleep.

### Conclusions

We examined sleep data from more than 2 million nights of data captured during a 4-year period among users of a popular sleep tracker in New York City. Our findings show women slept longer and demonstrated higher sleep quality than men, and that teenagers demonstrated abysmal rates of adherence to sleep duration recommendations. We also showed that sleep quality and duration vary seasonally. We also demonstrated visually that insufficient sleep is observed in greater prevalence among users located in outer boroughs of New York City. Future research may consider the role of sleep tracking for improving motivation to adhere to recommended sleep routines, such as consistent bedtime schedules. Future research may also examine the role of sleep tracking and health profiles of users over time.

## Appendix

Multimedia Appendix 1 Descriptive statistics of sleep variables, including duration and quality (n=2,161,067 nights of sleep tracking).

Multimedia Appendix 2 Sleep duration recommendations and sleep quality by age group (n=2,161,067 nights of sleep tracking).
