# Supplementary material for: Four-Year Trends in Sleep Duration and Quality: A Longitudinal Study Using Data from a Commercially Available Sleep Tracker
Source: J Med Internet Res. 2020 Feb 20;22(2):e14735. doi: 10.2196/14735 (PMC7059084; doi:10.2196/14735)
Supplement: Multimedia Appendix 1 [file jmir_v22i2e14735_app1.docx]

**Multimedia Appendix 1**

**Descriptive statistics of sleep variables, including duration and quality (n=2,161,067 nights of sleep tracking).**

|  |  | **Age** | | | **Duration** | | | **Quality** | | |
| --- | --- | --- | --- | --- | --- | --- | --- | --- | --- | --- |
| **Variable** |  | ***Mean*** | ***SD*** | ***P-value*** | ***Mean*** | ***SD*** | ***P-value*** | ***Mean*** | ***SD*** | ***Median*** |
| Total | 2,161,067 | 31.0 | 10.6 |  | 7.1 | 1.4 |  | 72.3 | 14.2 |  |
| Sex |  |  |  | <.001 |  |  | <.001 |  |  | <.001 |
| Female | 1,003,421 | 30.3 | 10.4 |  | 7.3 | 1.4 |  | 73.4 | 14.1 |  |
| Male | 1,157,646 | 31.6 | 10.7 |  | 7.0 | 1.3 |  | 71.3 | 14.2 |  |
| Weekend v. Weekday | |  |  | <.001 |  |  | <.001 |  |  | <.001 |
| Weekdays | 1,299,037 | 31.0 | 10.7 |  | 7.1 | 1.3 |  | 71.9 | 13.6 |  |
| Weekend | 862,030 | 31.1 | 10.6 |  | 7.2 | 1.5 |  | 72.8 | 15.0 |  |
| Age Group |  |  |  | <.001 |  |  | <.001 |  |  | <.001 |
| Teens | 97,156 | 15.8 | 1.2 |  | 7.2 | 1.5 |  | 72.4 | 14.9 |  |
| Young Adults | 739,423 | 22.9 | 2.4 |  | 7.1 | 1.4 |  | 71.7 | 14.6 |  |
| Adults | 1,298,200 | 36.0 | 8.6 |  | 7.2 | 1.3 |  | 72.5 | 13.9 |  |
| Older Adults | 26,288 | 69.5 | 3.9 |  | 7.4 | 1.4 |  | 74.1 | 14.1 |  |
