# Supplementary material for: Four-Year Trends in Sleep Duration and Quality: A Longitudinal Study Using Data from a Commercially Available Sleep Tracker
Source: J Med Internet Res. 2020 Feb 20;22(2):e14735. doi: 10.2196/14735 (PMC7059084; doi:10.2196/14735)
Supplement: Multimedia Appendix 2 [file jmir_v22i2e14735_app2.docx]

**Multimedia Appendix 2**

**Sleep duration recommendations and sleep quality by age group (n=2,161,067 nights of sleep tracking).**

|  |  | **Total** | **Female** | **Male** |
| --- | --- | --- | --- | --- |
| **Variable** |  | ***N(%)*** | ***N(%)*** | ***N(%)*** |
| **Teens [13,17]** |  |  |  |  |
| **Sleep Duration** | Not appropriate [0,7] | 40,064(41.2) | 216,558(39.1) | 18,406(44.1) |
|  | May be appropriate [7,8] | 28,928(29.8) | 16,276(29.4) | 12,652(30.3) |
|  | Recommended [8,10] | 25,491(26.2) | 15,668(28.3) | 9,823(23.5) |
|  | May be appropriate [10,11] | 1,919(2.0) | 1,288(2.3) | 631(1.5) |
|  | Not appropriate [11,24] | 754(0.8) | 499(0.9) | 255(0.6) |
| **Sleep Quality** | Good | 20,951(21.6) | 12,824(76.8) | 8,127(19.5) |
|  | Poor | 76,205(78.4) | 42,565(23.2) | 33,640(80.5) |
| **Young Adults [18,25]** |  |  |  |  |
| **Sleep Duration** | Not appropriate [0,6] | 142,494(19.3) | 61,372(17.1) | 81,122(21.3) |
|  | May be appropriate [6,7] | 183,152(24.8) | 81,237(22.7) | 101,915(26.8) |
|  | Recommended [7,9] | 364,065(49.2) | 185,163(51.6) | 178,902(47.0) |
|  | May be appropriate [9,10] | 37,856(5.1) | 23,071(6.4) | 14,785(3.9) |
|  | Not appropriate [10,24] | 11,856(1.6) | 7,741(2.2) | 4,115(1.1) |
| **Sleep Quality** | Good | 140,428(19.0) | 76,868(21.4) | 63,560(16.7) |
|  | Poor | 598,995(81.0) | 281,716(78.6) | 317,279(83.3) |
| **Adults [26,64]** |  |  |  |  |
| **Sleep Duration** |  |  |  |  |
|  | Not appropriate [0,6] | 217,308(16.7) | 82,599(14.3) | 134,709(18.7) |
|  | May be appropriate [6,7] | 330,459(25.5) | 132,118(22.8) | 198,341(27.6) |
|  | Recommended [7,9] | 673,947(51.9) | 319,403(55.2) | 354,544(49.3) |
|  | May be appropriate [9,10] | 59,004(4.5) | 34,032(5.9) | 24,972(3.5) |
|  | Not appropriate [10,24] | 17,482(1.3) | 10,524(1.8) | 6,958(1.0) |
| **Sleep Quality** | Good | 249,970(19.3) | 127,466(22) | 122,504(17) |
|  | Poor | 1,048,230(80.7) | 451,210(78) | 597,020(83) |
| **Older Adults [65,84]** |  |  |  |  |
| **Sleep Duration** | Not appropriate [0,5] | 1241(4.7) | 471(4.4) | 770(5.0) |
|  | May be appropriate [5,7] | 8,363(31.8) | 3,148(29.2) | 5,215(33.6) |
|  | Recommended [7,8] | 8,914(33.9) | 3,912(36.3) | 5,002(32.2) |
|  | May be appropriate [8,9] | 5,280(20.1) | 2,188(20.3) | 3,092(19.9) |
|  | Not appropriate [9,24] | 2,490(9.5) | 1,053(9.8) | 1,437(9.3) |
| **Sleep Quality** | Good | 6,260(23.8) | 2,508(23.3) | 3,752(24.2) |
|  | Poor | 20,028(76.2) | 8,264(76.7) | 1,1764(75.8) |
